# Grb7 Upregulation Is a Molecular Adaptation to HER2 Signaling Inhibition Due to Removal of Akt-Mediated Gene Repression

**DOI:** 10.1371/journal.pone.0009024

**Published:** 2010-02-02

**Authors:** Alessio Nencioni, Michele Cea, Anna Garuti, Mario Passalacqua, Lizzia Raffaghello, Debora Soncini, Eva Moran, Gabriele Zoppoli, Vito Pistoia, Franco Patrone, Alberto Ballestrero

**Affiliations:** 1 Department of Internal Medicine, Centre of Excellence for Biomedical Research, University of Genoa, Genoa, Italy; 2 Department of Experimental Medicine-Biochemistry Section, Centre of Excellence for Biomedical Research, University of Genoa, Genoa, Italy; 3 Italian Institute of Biostructures and Biosystems (INBB), Rome, Italy; 4 Laboratory of Oncology, Department of Experimental and Laboratory Medicine, G. Gaslini Institute, Genova, Italy; Roswell Park Cancer Institute, United States of America

## Abstract

The efficacy of anti-HER2 therapeutics, such as lapatinib and trastuzumab, is limited by primary and acquired resistance. Cellular adaptations that allow breast cancer cell to survive prolonged HER2 inhibition include de-repression of the transcription factor FOXO3A with consequent estrogen receptor activation, and/or increased HER3 signaling. Here, we used low-density arrays, quantitative PCR, and western blotting to determine how HER2 signaling inhibition with lapatinib or PI3K inhibitors affects the expression of genes involved in breast cancer metastatic spread and overall prognosis. Retroviral transgenesis was used to express constitutively active forms of Akt in the HER2^+^ breast cancer cell line SKBR3, and Grb7 in MCF7 cells. Specific gene silencing was obtained by siRNAs transfection. A murine BT474 xenograft cancer model was used to assess the effect of lapatinib on gene expression *in vivo*. We found that lapatinib induces upregulation of Grb7, an adaptor protein involved in receptor tyrosine kinase signaling and promoting cell survival and cell migration. Grb7 upregulation induced by lapatinib was found to occur in cancer cells *in vitro* and *in vivo*. We demonstrate that Grb7 upregulation is recreated by PI3K inhibitors while being prevented by constitutively active Akt. Thus, Grb7 is repressed by PI3K signaling and lapatinib-mediated Akt inhibition is responsible for Grb7 de-repression. Finally, we show that Grb7 removal by RNA-interference reduces breast cancer cell viability and increases the activity of lapatinib. In conclusion, Grb7 upregulation is a potentially adverse consequence of HER2 signaling inhibition. Preventing Grb7 accumulation and/or its interaction with receptor tyrosine kinases may increase the benefit of HER2-targeting drugs.

## Introduction

HER2/ERBB2/neu is amplified in 20–30% breast cancers where it acts as a driving oncogene conferring propensity to metastasize and adverse prognosis [1]. HER2 amplification leads to increased HER2 levels at the cell surface resulting in aberrant signaling from HER2-containing homo- and hetero-dimers [2]. HER2 oncogenic properties are thought to result from increased activation of the signaling cascades downstream of HER2. Particularly, signaling through the Ras-MAPK cascade is responsible for cell proliferation, cell migration, and angiogenesis, while the phosphoinositide 3-kinase (PI3K)-Akt pathway exerts multiple anti-apoptotic and growth promoting effects [2].

HER2-targeting therapeutics, such as the monoclonal antibody trastuzumab (Herceptin) and the dual HER2/EGFR tyrosine kinase inhibitor lapatinib (Tykerb), are successfully applied to the treatment of patients with HER2^+^ breast cancer [3], [4], [5]. In addition, recent data indicate that these drugs could potentially also find applications in cancers of different histology [6], [7]. However, the efficacy of anti-HER2 therapeutics is restricted to a fraction of the patients, and even in those that initially respond to treatment, acquired resistance is commonly observed [8], [9].

Chronic exposure to inhibitors of HER-family proteins results in cellular adaptations with the potential of reducing the efficacy of these drugs. Sergina at al. described a feedback mechanism controlled by Akt whereby Akt inhibition in response to HER2 tyrosine kinase inhibitors leads to HER3 redistribution to the cell membrane and to sustained HER3 phosphorylation [10]. Increased HER3 signaling would in turn then restore Akt activity and thus lead to drug resistance. Similarly, prolonged Akt inhibition in lapatinib-treated cells was shown to de-repress FOXO3A, a transcription factor that up-regulates estrogen receptor (ER) signaling [9]. As a result, breast cancer cells that would previously rely on HER2 signaling for survival, become supported by ER activity and can thereby escape cell death as a consequence of HER2 inhibition. Finally, acquired resistance to trastuzumab in an experimental model was found to correlate with increased EGFR, EGF, TGF-α, and heregulin levels [11]. Overall, a detailed understanding of the molecular consequences of HER2 inhibition appears to be essential for the design of therapeutic strategies to overcome resistance and improve clinical outcomes.

Here, we used low-density arrays (LDAs) to assess how HER2 tyrosine kinase inhibition by lapatinib would affect the expression of a panel of genes involved in breast cancer biology and metastatic behavior. We show that lapatinib causes rapid upregulation of Grb7, an adaptor protein that is typically co-amplified with HER2, participates in HER2 signaling, and promotes cell survival and cell migration. We identify a negative feedback loop mediated by Akt as responsible for repressing Grb7. Finally, we show that preventing Grb7 accumulation by RNA-interference potentiates the activity of lapatinib.

## Materials and Methods

### Cells and Reagents

SKBR3, BT474, MCF7, MDA-MB231, and Phoenix cells were all obtained from ATCC. Cell were grown in RPMI medium (Sigma-Aldrich Italia, Milan, Italy), supplemented with 10%FBS, L-glutamine and antibiotics. Lapatinib was generously provided by GlaxoSmithKline. LY294002, wortmannin, puromycin, G418, and protamine sulfate, were purchased from Sigma-Aldrich (Sigma Aldrich Italia, Milan, Italy).

### Viability Assays and Cell Cycle Analysis

5×10^3^ cells/well were seeded in 96 well plates in medium containing 1% FBS. Cells were allowed to adhere for 24 h and were subsequently incubated with the indicated drug concentrations. Each condition was tested in triplicate wells. Viability was assessed 120 h later by Celltiter96 Aqueous1 (Promega Italia, Milan, Italy) using a standard ELISA reader. Specific death was calculated with the following formula: 100-[(x-background signal)/(untreated-background signal)x1.

For cell cycle analysis, adhering cells were resuspended in a buffer containing 0.1% Na citrate, 0.1% Triton-X, and 50 µg/ml propodium iodide. Thereafter, the isolated cell nuclei were analyzed by flow cytometry using a FACS Calibur (Becton Dickinson, Milan, Italy).

### Microscopy

Cells were imaged at room temperature using with the 40X magnification of a Zeiss AXIOVERT200 microscope, an Olympus C-4040ZOOM camera, and the software Olympus CAMEDIA Master 2.5.

### Immunostaining

Adhering cells were harvested, washed, and incubated for 15′ at room temperature with a FITC-conjugated anti-TFRC (CD71) antibody (Becton Dickinson), or a FITC-conjugated anti-EGFR antibody (sc120-FITC, Santa Cruz Biotechnology, Heidelberg, Germany). Thereafter, cells were analyzed by flow cytometry on a FACS Calibur.

### Retroviral Transgenesis

The plasmids MSCVpuro-wild type (WT) Akt and MSCVpuro-Akt S473D [12] were a gift of Dr. Sabatini (Whitehead Institute for Biomedical Research, Cambridge, MA, USA). pJP1520 and pJP1520-Grb7 were purchased from the Dana-Farber/Harvard Cancer Center DNA Resource Core (Boston, MA, USA). 1.5×10^6^ Phoenix cells were plated in 4 ml medium in 6 cm-dishes and allowed to adhere for 24 h. Thereafter, cells were transfected with 4 µg plasmid DNA using Transit 293 (Mirus Bio, Madison, WI, USA) according to the manufacturer's instructions. The viral supernatant was harvested 36 and 48 h later and used to infect SKBR3 or MCF7 cells in 6 cm dishes in the presence of 5 µg/ml protamine sulfate. Successfully infected cells were selected using 1.5 µg/ml puromycin.

### siRNA Transfection

Grb7 siRNAs (ON-TARGET-Plus Smart Pool) and the respective non targeting control siRNAs were purchased from Dharmacon (ThermoFisher Scientific Inc., Euroclone, Milan, Italy). 10^5^ cells/well were plated in 12-well plates, allowed to adhere for 48 h, and then transfected using Dharmafect (Dharmacon) according to the manufacturer's instruction. Grb7 silencing was verified by immunoblotting and Q-PCR.

### Transient Transfections

2×10^5^ SKBR3 cells were plated in 6-well plates and allowed to adhere for 24 h. Thereafter, cell were transfected with 2 µg plasmid DNA using Lipofectamine (Invitrogen Italia Srl, Milan, Italy) according to the manufacturer's instructions. The plasmids pcDNA3 FOXO1A, pcDNA3 FOXO1A AAA (T24A, S256A, S319A), pcDNA3 FOXO3A, pcDNA3 FOXO3A AAA (T32A, S253A, and S315A) were purchased from Addgene (Cambridge, MA, USA). pcDNA3 was a king gift of Dr. Alberto Inga (National Cancer Institute, Genoa, Italy). Thereafter, cells were cultured with 500 µg/ml G418 for two weeks before being used for protein lysates preparation.

### LDAs and Quantitative Real-Time PCR (Q-PCR)

Total RNA was isolated from cell lysates using QIAGEN RNeasy Mini anion-exchange spin columns (QIAGEN, Hilden, Germany) according to the manufacturer's instructions. Total RNA (1 µg) was reverse-transcribed using SuperScript RTII (Invitrogen, Karlsruhe, Germany) and oligo(dT) as primers. For LDAs, a primer collection for a panel of 96 genes selected for their relevance in breast cancer prognosis and metastatic behaviour (Table S1) was assembled by Applied Biosystems (Carlsbad, CA, USA). LDAs and Q-PCRs were performed on a 7900HT Fast Real Time PCR System (Applied Biosystems). Q-PCRs for GRB7, GRB2, and RPLP0 were performed with pre-designed primers by Applied Biosystems. Results were normalized to RPLP0 expression and plotted as mRNA fold induction in drug-treated versus vehicle-treated cells using the 2^-ΔΔCT^ method [13].

### Immunoblotting

Cell lysates were prepared by resuspending cells in SDS sample buffer [6.25 mol/L Tris-HCl (pH 6.8), 2% SDS, 10% glycerol, 2% β-mercaptoethanol, 0.005% bromophenol blue; Boston Bioproducts, Boston, MA]. Cell lysates were boiled at 100°C for 10 minutes and stored at −20°C for subsequent use. Proteins were separated on a SDS-polyacrylamide gel and electroblotted to a polyvinylidene fluoride membrane (Pall Gelman Laboratory, Ann Arbor, MI). Proteins were visualized by probing the membranes with the following antibodies: anti-phospho Akt (Ser473), anti-Akt, anti-HER2 (Cell Signaling Technology, Euroclone, Milan, Italy), anti-Grb7 (N-20, Santa Cruz Biotechnology), anti-FOXO3a (rabbit polyclonal, Millipore, Billerica, MA, USA), and γ-tubulin (Sigma Aldrich). Standard enhanced chemiluminescence was used for protein bands detection.

### Co-Immunoprecipitation

Cells (3×10^6^) were washed twice with ice-cold PBS and then scraped off the plates in cell-lysis buffer (1% NP40, 20 mM TRIS/HCl pH 7.4, 0.14 M NaCl, 1mM EDTA, 1mM EGTA) containing protease inhibitor cocktail and phosphatase inhibitor cocktail I & II (Sigma-Aldrich). Lysates were prepared after 15 min of incubation on ice by repeated pipetting and centrifugation in a microfuge at 12,000 g for 10 min at 4°C. For coimmunoprecipitations, a anti-HER2 antibody (Cell Signaling) was covalently coupled to protein G Dynabeads (2 µg of antibodies per 30 µl beads) using BS^3^ (Bis[sulfosuccinimidyl]suberate; Pierce, Rockford, IL) according to the manufacturer's specification, and then incubated with 1 mg of cell extract supernatant for 3 h at 4°C. The immunoprecipitates were washed five times with lysis buffer, eluted with 25 µl of electrophoresis sample buffer, and heated at 95°C for 5 min. Thereafter, proteins were separated by immunoblotting, transferred to PVDF membranes and revealed using appropriate antibodies and standard enhanced chemiluminescence.

### Tumor Xenografts in Nude Mice

This study was performed in compliance with the US Department of Health and Human Services Guide for the Care and Use of Laboratory Animals and approved by the Internal Review Board of the Advanced Biotechnology Center (ABC) in Genoa, Italy. Six- to eight-week-old female BALB/c athymic mice (nu+/nu+, n = 8) were acquired from Charles Rivers Laboratories. A 17 β-estradiol pellet (Innovative Research of America) was inserted s.c. to each mouse 1 d before injection with BT474 cells. 2×10^7^ BT474 cells were injected s.c., and treatment was initiated when the tumors appeared as established palpable masses (∼3 weeks after the injection). 50 mg/kg body weight lapatinib was given daily for three days by oral gavage in 0.5% hydroxypropylmethycellulose and 0.1% Tween 80. Thereafter, mice were anesthetized and killed by cervical dislocation. Tumors were divided in two parts and stored in RNAlater (Ambion-Applied Biosystems, Milan, Italy) for subsequent RNA extraction, and in formalin for histological examination (hematoxylin-eosin) and HER2 immunohistochemistry (HercepTest, Dako, Carpinteria, CA).

### Promoter Analysis

Promoter analysis of the Grb7 gene was performed by Genomatix software (Genomatix Software Inc., Ann Harbor, MI, USA).

### Statistical Analysis

Each experiment was performed at least three times with similar results. Unpaired *t*-tests were performed to evaluate the significance of the results.

## Results

### Gene Expression Modulation in Response to Lapatinib

**Figure 1:**
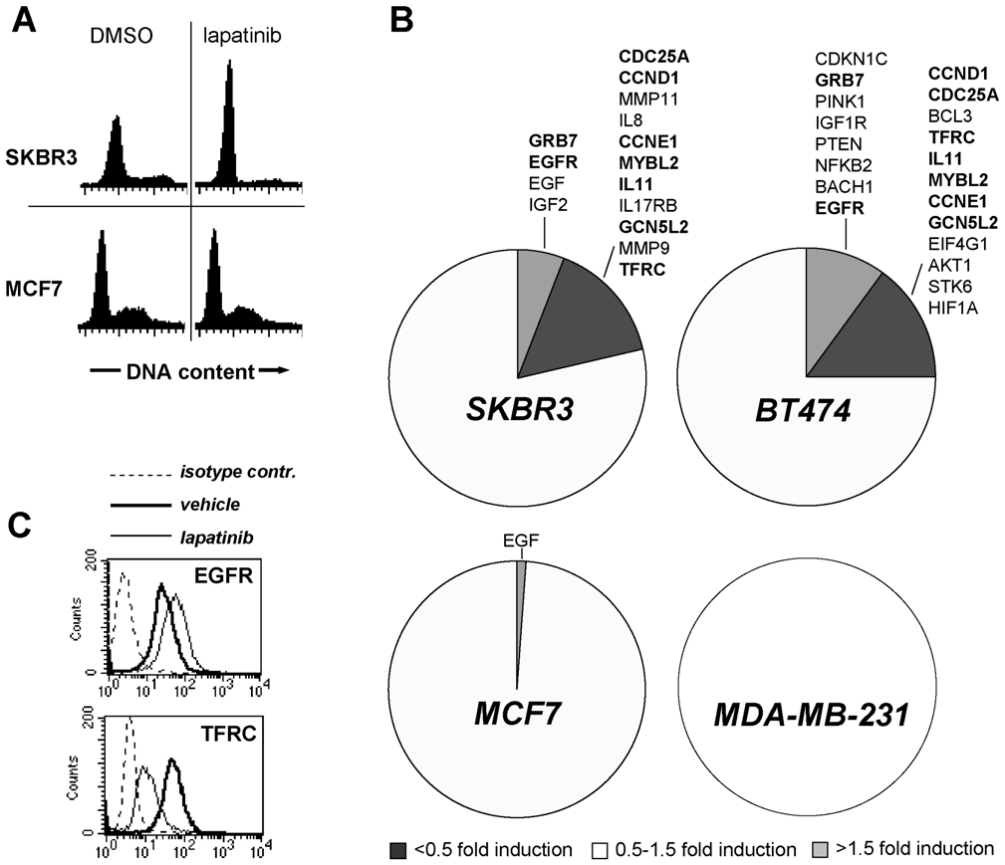
Changes in gene expression in breast cancer cell lines in response to lapatinib. A, B, 1×10^6^ cells were seeded in 10-cm culture dishes, allowed to adhere for 24 h and subsequently treated for 12 h with 300 nM lapatinib or vehicle DMSO. Thereafter, cells were harvested, washed and used for cell cycle analysis (A) or for RNA extraction. Subsequently, LDAs were performed to compare gene expression in lapatinib- vs. vehicle-treated cells (B). C, 2×10^5^ SKBR3 cells/well were seeded in 6-well plates, allowed to adhere for 24 h and subsequently treated for 36 h with 300 nM lapatinib or vehicle DMSO. Thereafter, TFRC/CD71 and EGFR expression were determined by flow cytometry. B, results were calculated as means of four separate experiments. A, C one representative experiment out of four is shown.

We were interested in assessing the repercussions of HER2 signaling inhibition on a panel of genes involved in breast cancer aggressiveness and metastasis (a full list of the genes and of the TLDA results is appended as Table S1). To this end, we made use of HER2-overexpressing (SKBR3 and BT474) and non-HER2-overexpressing (MCF7 and MDA-MB-231) breast cancer cell lines. According to previous evidence that lapatinib activity correlates with the degree of HER2 expression [14], we found that SKBR3 and BT474 cells underwent G1 cell cycle arrest, and subsequently died in response to this drug (Figure 1A and data not shown). *Vice versa*, in MCF7 and MDA-MB-231 cells lapatinib showed no effect on cell cycle and viability for concentrations up to 1 µM (Figure 1A and data not shown). SKBR3, BT474, MCF7 and MDA-MB-231 cells were treated with lapatinib (300 nM) and the gene expression profiles of lapatinib- versus vehicle-treated cells were determined in low-density arrays. Lapatinib showed a much greater impact on the gene expression profile of SKBR3 and BT474 cells as compared to the two non-HER2-overexpressing cell lines MCF7 and MDA-MB-231 (Figure 1B and Table S1). In Figure 1B, we arbitrarily chose <0.5 fold induction and >1.5 fold induction to define those genes that were down- and up-regulated by drug treatment, respectively. Applying this criterion, in SKBR3 cells, 6% and 16% of the examined genes were up- and down-regulated by lapatinib, respectively. In BT474, 10% of the genes were upregulated while 15% were upregulated. In sharp contrast, in MCF7 cells only one gene out of eighty two was modulated by lapatinib (EGF, upregulated), while in MDA-MB-231 no gene up- or down-regulation was detected according to the previously defined criterion.

Importantly, several of the observed modifications in gene expression were known or predictable based on the mode of action of lapatinib. For instance, CCND1, CCNE1, and CDC25, which we found to be down-regulated by lapatinib in SKBR3 and BT474 cells, are all involved in the G1/S transition phase of the cell cycle. Thus, these changes paralleled and likely accounted for the G1 cell cycle arrest observed in response to lapatinib. Moreover, CCND1 downregulation has been documented in primary tumors from patients treated with this drug [15]. Among the other genes that were consistently down-regulated by lapatinib in HER2-overexpressing cells, GCN5L2 is a still poorly characterized histone deacetylase whose expression was found to correlate with ERα in breast cancer [16]. MYBL2 is a transcription factor whose expression correlates with the risk of relapse in node negative breast cancer [17]. Moreover, MYBL2 was also found to be enriched in high-grade ER-negative tumors and to associate with stem or progenitor function, and with cancer cell proliferation [18]. Interestingly, in SKBR3 and BT474, lapatinib consistently down-regulated the transferrin receptor TFRC/CD71 (Figure 1B, C). Since TFRC expression is promoted by the Akt/mTOR pathway [19], [20], this finding is in line with lapatinib-mediated Akt inhibition in HER2-overxpressing breast cancer cells [15].

We observed that a fraction of the tested genes was upregulated by lapatinib in HER2-overexpressing cells. For some of these changes, it was appealing to speculate that they may result from feedback loops involving gene de-repression, and be aimed to compensate for HER2 signaling inhibition. Grb7 and EGFR were consistently upregulated in SKBR3 and in BT474 cells (Figure 1B, C). Increased EGFR levels have previously been reported in response to erlotinib, a small molecule EGFR inhibitor, and to trastuzumab [11], [21]. Using EGFR-specific siRNAs or EGFR-targeted drugs was proposed as a strategy to counter this adaptation and induce tumor regression [11], [21].

Here, we focused on Grb7 because of its emerging relevance in breast cancer prognosis, and its supposed role in anticancer drug resistance [17], [22], [23]. Grb7 is an adaptor protein participating in signaling downstream of receptor tyrosine kinases (RTKs) [24], [25], [26], [27]. Grb7 also plays a role in integrin signaling and in cell migration through its interaction with focal adhesion kinase (FAK) [28], [29], [30], [31]. Interestingly, Grb7 is located on the HER2 amplicon, is co-amplified and co-overexpressed with HER2 in cancer, and physically interacts with HER2, HER3, and HER4 [26], [32], [33]. Studies in animal models of HER2-driven tumorigenesis confirmed that Grb7 and HER2 are usually co-amplified, and a strong correlation between HER2, phospho-HER2, and Grb7 copy number and protein levels was detected [34], [35]. Thus, Grb7 and HER2, at least when amplified and overexpressed, appear act in concert to drive breast cancer formation.

### Grb7 Is Repressed by the PI3K-Akt Pathway

**Figure 2:**
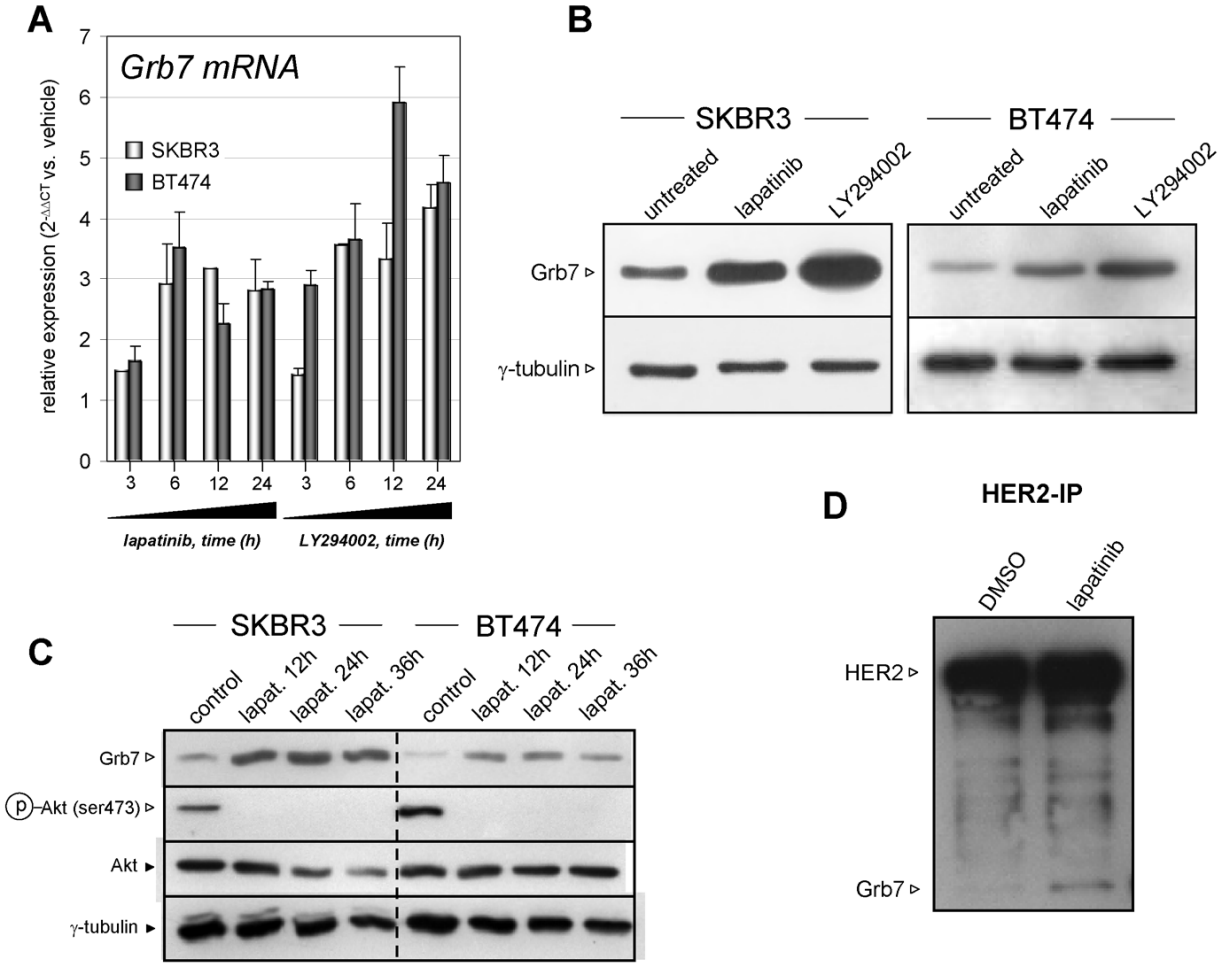
Lapatinib and the PI3K inhibitor LY294002 induce Grb7 upregulation. A, 2×10^5^ SKBR3 or BT474 cells/well were seeded in 6-well plates, allowed to adhere for 24 h and subsequently treated with 300 nM lapatinib or 20 µM LY294002 for the indicated times. Thereafter, total RNA was isolated, and Grb7 mRNA levels were compared to those in vehicle-treated cells. B, C, 2×10^5^ SKBR3 or BT474 cells/well were seeded in 6-well plates and allowed to adhere for 24 h. Thereafter, cells were treated with 300 nM lapatinib or 20 µM LY294002 for 24 h and then used for protein lysates preparation (B); or incubated with 300 nM lapatinib for the indicated times before being lysed. Grb7, γ-tubulin (B, C), phospho-Akt, and total Akt (C) levels were determined by immunoblotting. D, 1.5×10^3^ SKBR3 cells were plated in 10 cm dishes, allowed to adhere, and treated with 300 nM lapatinib for 36 h. Thereafter, cells were resuspended in lysis buffer and HER2 was immunoprecipitated using a specific anti-HER2 antibody. The immunoprecipitated protein was separated by SDS PAGE, blotted onto a PVDF membrane and HER2 and Grb7 were detected using appropriate antibodies. A, Results are presented as means ± SD of three separate experiments. B-D, One representative experiment out of three is shown.

Akt is involved in several forms of adaptations and feedback loops responsible for modulating RTK signaling [9], [10]. We therefore hypothesized that Grb7 upregulation as a consequence of HER2 and EGFR tyrosine kinase inhibition would reflect inactivation of the PI3K-Akt signal transduction cascade. In line with this hypothesis, we found that lapatinib and the PI3K inhibitor LY296004 both caused rapid upregulation of Grb7 mRNA in SKBR3 and BT474 cells (Figure 2A). Changes in Grb7 mRNA translated into a striking increase in Grb7 protein levels in response to lapatinib, LY294002, and wortmannin, another PI3K inhibitor (Figure 2B and data not shown). Figure 2C shows a time course experiment where Grb7 protein and Akt phosphorylation (Ser473) are monitored over time in response to lapatinib. Grb7 upregulation appears to be a relatively early event, being well detectable already after a 12 h treatment. Interestingly, in co-immunoprecipitation experiments, we found that Grb7-HER2 physical interaction is maintained also in lapatinib treated cells (Figure 2D).

**Figure 3:**
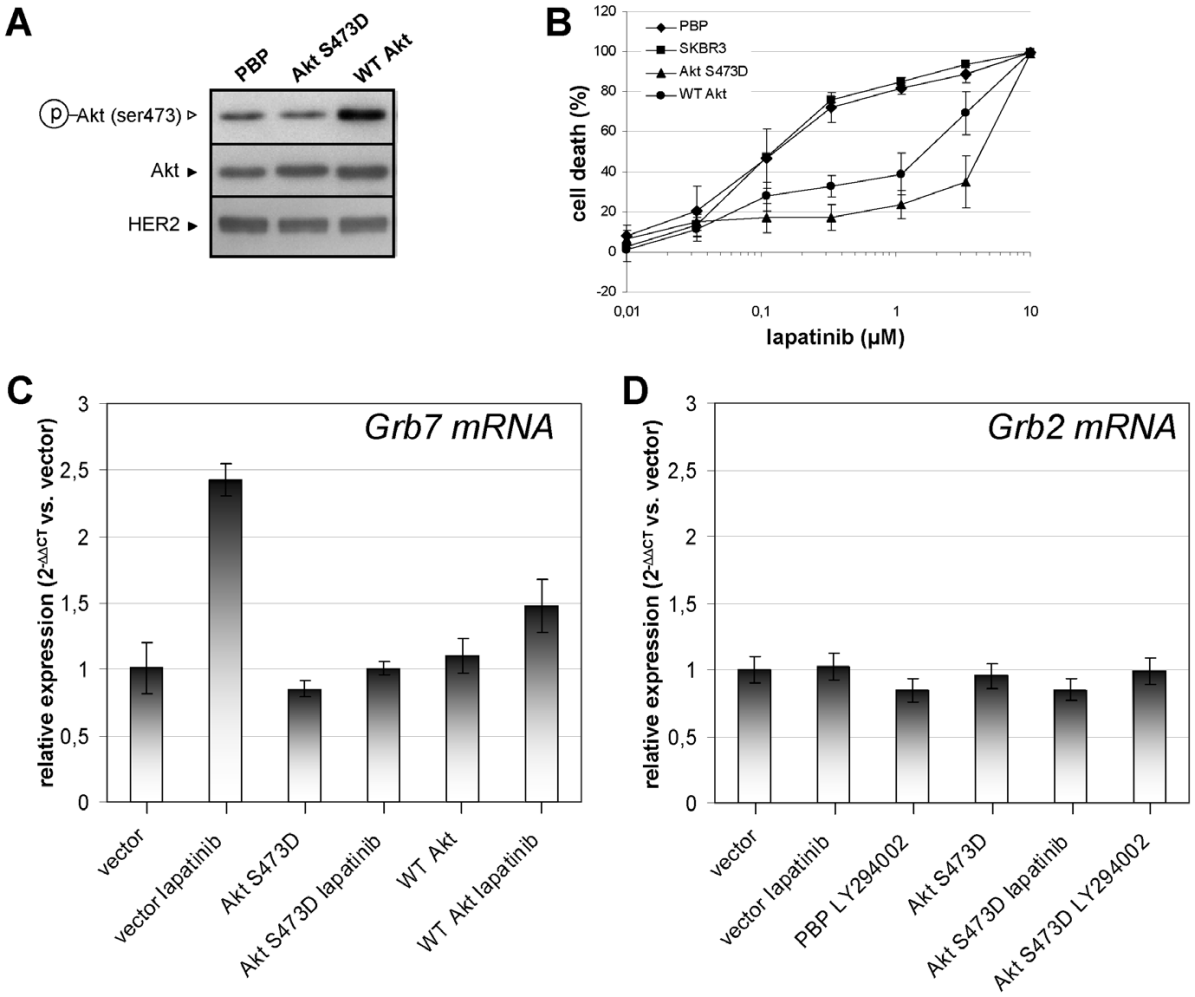
Active Akt prevents Grb7 upregulation in response to lapatinib. A-D, SKBR3 cells were transduced with empty MSCV (vector), MSCVpuro-Akt S473D, or MSCVpuro-WT Akt. A, 2×10^5^ cells/well were plated in 6-well dishes, allowed to adhere for 24 h and subsequently used for cell lysates preparation. Phospho-Akt, total Akt and HER2 levels were detected by immunoblotting. B, 5×10^3^ cells/well were plated in 96-well plates, allowed to adhere and subsequently treated with lapatinib at the indicated concentrations. Viability was assessed 5 days later by colorimetric assay. C, D, 2×10^5^ cells/well were plated in 6-well dishes, allowed to adhere for 24 h and then incubated with or without 300 nM lapatinib (C, D) or 20 µM LY294002 for 24 h (D). Thereafter, total RNA was isolated and Grb7 (C) and Grb2 (D) mRNA levels were compared to those in vector cells. B-D Results are shown as means ± SD of three independent experiments. A, one representative experiment out of three is presented.

To confirm the involvement of Akt inhibition in Grb7 upregulation via lapatinib, we engineered SKBR3 cells to express a constitutively active Akt isoform (Akt S473D) or overexpress a WT Akt allele [12]. While WT Akt overexpression led to increased phospho-Akt levels (Ser473), this effect was not detected with the mutant isoform, possibly due to conformational changes in the antibody-binding site as a consequence of the mutation itself (Figure 3A). Nonetheless, both alleles increased cell size in MCF7 cells (data not shown) and reduced susceptibility to lapatinib in SKBR3 cells (Figure 3B). Expression of either Akt S473D or WT Akt prevented Grb7 upregulation in response to lapatinib, confirming that active Akt represses Grb7 transcription (Figure 3C). Finally, we investigated whether this type of regulatory mechanism would apply to Grb2, another adaptor protein implicated in RTK signaling [36]. In this case, no Grb2 modulation in response to the pharmacological treatments was observed, nor did constitutively active Akt have any effect on Grb2 mRNA levels (Figure 3D). Thus, PI3K-mediated control does not appear to indiscriminately act on all HER interaction partners.

Akt is responsible for inhibiting the forkhead box-O (FOXO) transcription factors by phosphorylating them on several residues and thereby inducing their sequestration in the cytoplasm by 14-3-3 proteins [9]. FOXO3A re-activation as a consequence of Akt inhibition by lapatinib was shown to be responsible for increased ER transcription and, thereby, for acquired resistance to lapatinib itself [9]. Thus, we speculated that FOXO transcription factors could also be involved in the increased Grb7 transcription observed in response to lapatinib. Indeed, we detected several putative FOXO consensus binding sites in the Grb7 promoter (102–118, 207–223, 307–323, 336–352, 338–354, 487–503). To evaluate a potential role of FOXO3A in Grb7 expression, we engineered SKBR3 cells to overexpress a wild type FOXO3A allele or a FOXO3A isoform in which all the relevant phosphorylation sites are mutated rendering it constitutively active (FOXO3A AAA) [37]. However, neither of these modifications resulted in increased Grb7 expression (Figure S1). Similar results were obtained by overexpressing FOXO1A and its constitutively active isoform (FOXO1A AAA) (data not shown) [38]. Therefore, Grb7 upregulation in response to Akt inhibition appears to be independent of FOXO3A or FOXO1A.

### Grb7 Upregulation in Cancer Cells via Lapatinib Occurs *In Vivo*

**Figure 4:**
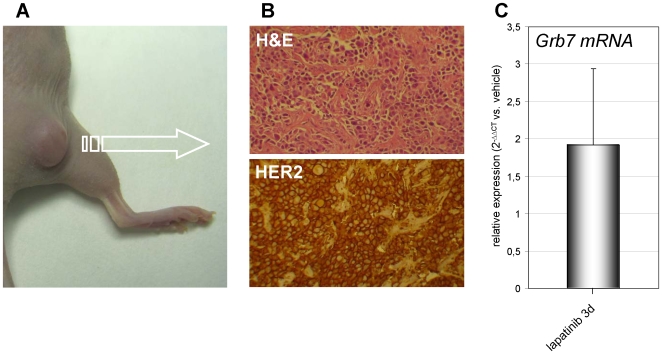
Lapatinib induces Grb7 upregulation in BT474 cells *in vivo*. A, B, BT474 tumor xenograft-bearing mice were given 50 mg/kg body weight lapatinib (n = 4) or vehicle DMSO (n = 4) daily for three days. At day 4, mice were euthanized and tumors were extracted and used for histology/immunohistochemistry and for RNA isolation. B, BT474 xenograft histology (H&E) and HER2 expression as detected in a vehicle-treated mouse. C, Grb7 mRNA level in the tumors was determined by Q-PCR in lapatinib vs. vehicle-treated mice.

To assess whether Grb7 upregulation due to lapatinib would occur in cancer cells *in vivo*, we made use of a BT474 murine xenograft model (Figure 4A). Figure 4B shows the typical histological appearance of these tumors as well as the strong HER2 overexpression detected by immunohistochemistry. Mice with established BT474 tumor masses were treated with lapatinib (or vehicle) for three days. Thereafter, Grb7 mRNA in the tumors was quantified by Q-PCR. Indeed, lapatinib treatment upregulated Grb7 mRNA by about two folds, indicating that increased Grb7 levels are likely to be found in HER2^+^ tumors *in vivo* in response to this drug.

### Grb7 Silencing Increases the Efficacy of Lapatinib

**Figure 5:**
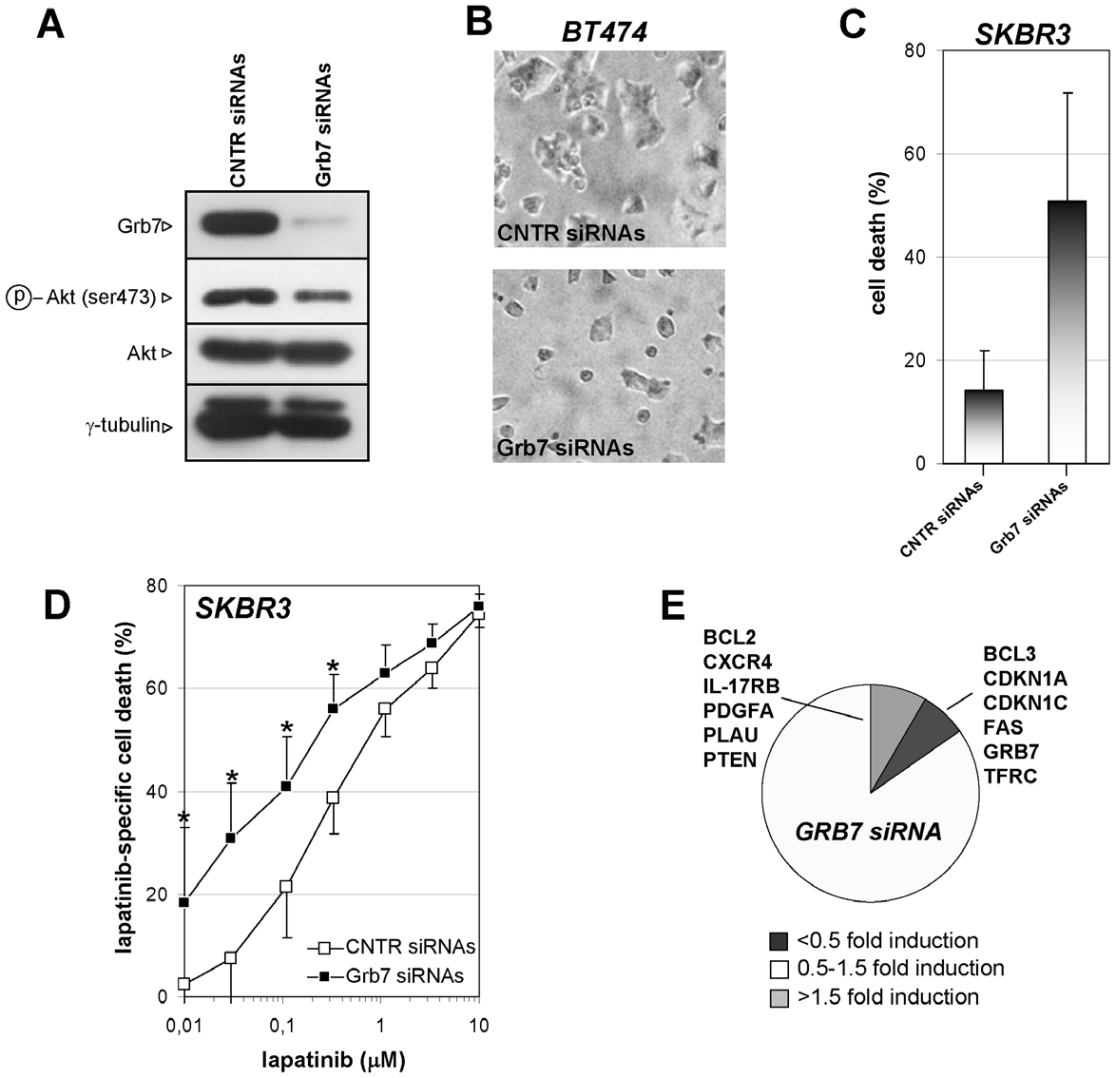
Grb7 silencing reduces cell viability and increases lapatinib efficacy. A-E, SKBR3 or BT474 cells were transfected with Grb7 siRNA or non-targeting control siRNA (CNTR siRNA). A, 2×10^5^ SKBR3 cells/well were seeded in 6-well plates, allowed to adhere for 48 h and subsequently used for protein lysates preparation. Grb7, phospho-Akt, total Akt and γ-tubulin levels were determined by immunoblotting. B, BT474 cells were transfected with Grb7 siRNA or CNTR siRNA and imaged by light microscopy five days later. C, D, 5×10^3^ siRNA-transfected or untransfected SKBR3/well were seeded in 96-well plates, allowed to adhere for 24 h and subsequently treated with or without lapatinib at the indicated concentrations. Five days later, viability was determined and compared to that of untransfected SKBR3 (C); lapatinib-specific death was determined by normalizing to baseline cell death of CNTR siRNAs- or Grb7 siRNAs-transfected SKBR3 (D). E, SKBR3 cells were transfected with Grb7 siRNA or CNTR siRNA. mRNA was extracted 3 days after transfection and gene expression was detected by LDAs. A, B, One representative experiment out of three is shown. C, D, Results are means ± SD of four independent experiments. E, Results were calculated as means of two separate experiments. *: p<0.05.

Grb7 promotes cell survival and increases cell proliferation [24], [39], [40]. Therefore, we sought to determine whether preventing Grb7 accumulation in response to lapatinib would improve the efficacy of this drug. To this aim, we silenced Grb7 using a pool of synthetic siRNAs that efficiently reduced Grb7 levels in the cells (Figure 5A). At the biochemical level, SKBR3 cells with silenced Grb7 showed reduced Akt phosphorylation, consistent with the notion that Grb7 participates in signal transduction downstream of HER2 [24]. In line with a recent report, Grb7 removal reduced cell viability in SKBR3 and BT474 cells. On the contrary, MCF7, that do not have HER2 and Grb7 amplification, and express very low Grb7 levels, were unaffected (Figure 5B, C, and data not shown) [41]. Finally, SKBR3 cells with silenced Grb7 expression were more susceptible to lapatinib for concentrations up to 300 nM (Figure 5D). Upon lapatinib concentrations >300 nM, the difference between SKBR3 cells with silenced Grb7 and control cells was no longer significant, possibly due to the pronounced cytotoxic activity of lapatinib alone.

**Figure 6:**
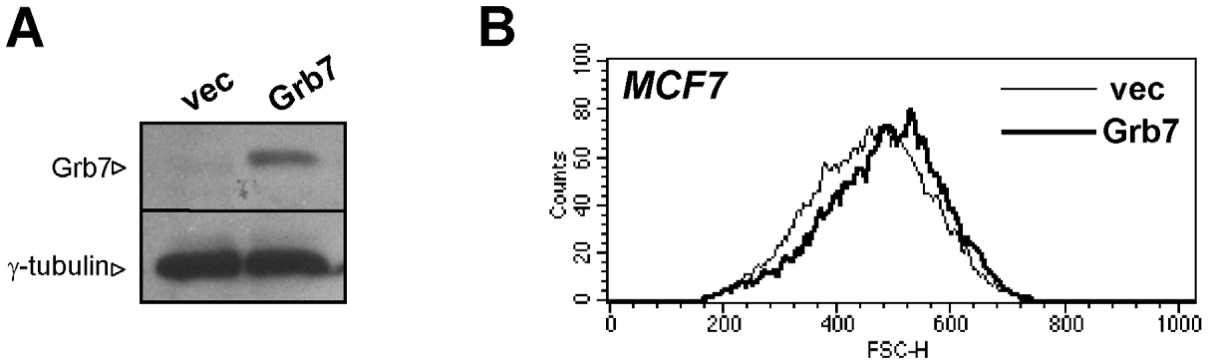
Grb7 overexpression increases cell size in MCF7 cells. MCF7 cells were transduced with Grb7 or with the respective empty vector (vec). A, 2×10^5^ cells/well were plated in 6-well plates, allowed to adhere and then used for cell lysate preparation. Grb7 and γ-tubulin levels were determined by immunoblotting. B, 10^6^ MCF7 cells engineered to express Grb7 or the respective vector control cells were cultured in 10-cm dishes and allowed to reach a 40–60% confluence. Subsequently, cells were harvested, washed and analyzed by flow cytometry. Histograms represent the cell forward scatter (FSC).

To gain insight into the mechanism whereby Grb7 inhibition/silencing affects cell viability and sensitizes cells to lapatinib, we performed cell cycle analysis and low-density arrays in SKBR3 cells with silenced Grb7. Reduced Grb7 levels did not have a major impact on the cell cycle profile (data not shown). On the other hand, similar to what observed with lapatinib, Grb7 removal decreased TFRC/CD71 expression, in line with a role for Grb7 in the HER2-Akt-mTOR pathway (Figure 5E). Finally, we overexpressed Grb7 in MCF7 cells, which normally express low levels of this protein (Figure 6A). Here, Grb7 expression would typically result in an increase in cell size (Figure 6B), which again is consistent with a role for this adaptor protein in pathways controlling cell growth and cell size such as the Akt-mTOR axis.

## Discussion

**Figure 7:**
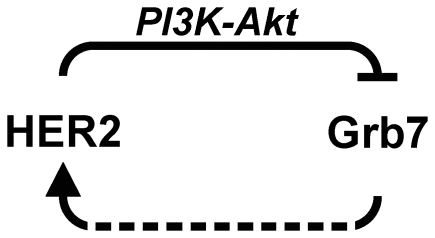
A feedback loop mediated by the PI3K-Akt axis controls Grb7 expression. Grb7 interacts with HER2, participates in HER2 signaling, and promotes cell survival and cell migration. HER2 exerts a repressive control on Grb7 via the PI3K-Akt pathway. Inhibition of HER2 signaling (e.g. by lapatinib) de-represses Grb7 causing its rapid upregulation. Reducing Grb7 with RNAi or preventing its interaction with HER2 using protein-protein interaction inhibitors may help increase the efficacy of anti-HER2 therapeutics and avoid the adverse consequences of Grb7 oncogenic activity.

In this study, we identify a functional interplay between HER2 and its interactor Grb7 whereby HER2 signaling represses Grb7 using the PI3K-Akt arm of its downstream signaling cascades. Inhibition of HER2 tyrosine kinase activity or of PI3K/Akt de-represses Grb7 causing its rapid upregulation (Figure 7). Noticeably, increased Grb7 expression appears to be independent of FOXO3A and FOXO1A re-activation in lapatinib-treated cells [9].

Our study reinforces the concept that adaptations involving gene de-repression and/or protein relocalization/posttranslational modification (as in the case of HER3) occur as a consequence of RTK inhibition and have the potential to reduce the benefit of RTK-targeting therapeutics [9], [10].

Grb7 acts as a pro-survival factor in breast cancer cells as shown by the fact that RNAi-mediated removal of this protein reduces cell viability. The mechanisms whereby Grb7 promotes cell survival are still unclear. Our data indicate a role for Grb7 in the HER2-Akt-mTOR pathway. Grb7 silencing reduces Akt activation and leads to TFRC/CD71 downregulation [19], [20]. Moreover, Grb7 overexpression in MCF7 cells increases their cell size. On the other hand, Grb7 may also owe its activity as a pro-survival factor to its interaction with other RTK or with other intracellular proteins [25], [27]. Finally, due to its participation in integrin signaling via FAK, Grb7 promotes cell migration [28], [29], [30], [31].

In line with its biological properties, Grb7 belongs to a group of genes conferring adverse prognosis in node-negative breast cancer [17]. In addition, Grb7 upregulation was shown to confer resistance to hormone therapy in breast cancer [22]. Acquired resistance to lapatinib and trastuzumab frequently occurs, possibly as a consequence of FOXO3A de-repression and increased ER signaling [8], [9]. It is conceivable that, in these conditions, Grb7 accumulation as a consequence of HER2 signaling inhibition may increase breast cancer cell aggressiveness and thereby speed up metastatic disease progression.

Our observation that Grb7 silencing increases lapatinib activity provides the proof of principle that interfering with this adaptor protein may be beneficial, although the mechanism(s) underlying this synergism is (are) not entirely clear. Grb7 upregulation *per se* does not seem to be sufficient to restore Akt phosphorylation (or Erk phosphorylation, data not shown) in the presence of lapatinib (at least in the incubation times tested in our experiments) regardless of a persistent interaction with HER2. Thus, Grb7 silencing is unlikely to cooperate with lapatinib by eliminating a residual Akt activity. *Vice versa*, it appears likelier that Grb7 reduction (also) affect other signaling pathways/intracellular processes whose obstruction increase susceptibility to HER2 inhibition.

RNAi-based therapeutics are eventually being developed and Grb7 siRNAs may thus potentially be coupled to anti-HER2 drugs [42]. Moreover, peptide inhibitors of Grb7-HER2 interaction are available and were previously shown to reduce proliferation and migration in various cancer cell lines [37], [43]. Combining these peptides with HER2-inhibiting drugs may help circumvent the negative effects of increased Grb7 levels.

In conclusion, Grb7 upregulation is a potentially adverse molecular side effect of HER2 signaling inhibition. Interfering with Grb7 accumulation may be advisable given its oncogenic activity and its capacity to increase the metastatic potential of a cell.

## Supporting Information

Table S1 Gene expression modulation by lapatinib in breast cancer cell lines. Supporting material. Gene list and LDA results.
(0.16 MB DOC)

Figure S1 FOXO3A does not affect Grb7 expression in SKBR3 cells. SKBR3 cells were transfected with plasmids encoding WT FOXO3A, FOXO3A AAA or the empty vector (pcDNA3) as a control. Cells were selected for 2 weeks using G418 before being used for cell lysates preparation. FOXO3A, Grb7, and γ-tubulin expression were detected by immunoblotting.
(0.70 MB TIF)
