# Supplementary material for: Grb7 Upregulation Is a Molecular Adaptation to HER2 Signaling Inhibition Due to Removal of Akt-Mediated Gene Repression
Source: PLoS One. 2010 Feb 2;5(2):e9024. doi: 10.1371/journal.pone.0009024 (PMC2814867; doi:10.1371/journal.pone.0009024)
Supplement: Table S1 — Gene expression modulation by lapatinib in breast cancer cell lines. Supporting material. Gene list and LDA results. (0.16 MB DOC) [file pone.0009024.s001.doc]

**Table S1. Gene expression modulation by lapatinib in breast cancer cell lines**

|  | *SKBR3* | *BT474* | *MDA-MB-231* | *MCF7* |
| --- | --- | --- | --- | --- |
| ***ABCC1-Hs00219905_m1*** | 1,044022 | 0,881542 | 0,932271 | 0,952962 |
| ***AKT1-Hs00178289_m1*** | 0,703788 | 0,4491 | 0,942683 | n.a.* |
| ***ATM-Hs00175892_m1*** | 1,48143 | 1,139178 | 0,863078 | n.a. |
| ***BACH1-Hs00230917_m1*** | 1,316357 | 1,532205 | 0,986157 | 1,128676 |
| ***BAG1-Hs00185390_m1*** | 0,871331 | 0,542754 | 0,933861 | 0,961927 |
| ***BARD1-Hs00184427_m1*** | 0,636386 | 0,511533 | 0,884991 | 1,182124 |
| ***BCL2-Hs00236808_s1*** | 0,62686 | n.a. | 1,19041 | 0,9624 |
| ***BCL2L11-Hs00708019_s1*** | 1,338645 | 1,070993 | 0,889443 | 0,948383 |
| ***BCL2L1-Hs00169141_m1*** | 1,121093 | 1,088798 | 0,965947 | 0,86344 |
| ***BCL2L2-Hs00187848_m1*** | 1,062931 | n.a. | 1,031664 | 1,22239 |
| ***BCL3-Hs00180403_m1*** | 0,637091 | 0,26281 | 0,845613 | 1,088035 |
| ***BIRC2-Hs00236911_m1*** | 1,179101 | 0,531113 | 1,112449 | 1,220378 |
| ***BIRC3-Hs00154109_m1*** | 1,130375 | 0,650632 | 0,983322 | 0,876429 |
| ***BIRC5-Hs00153353_m1*** | 0,715323 | 0,582251 | 1,059764 | 1,02107 |
| ***BTRC-Hs00182707_m1*** | n.a. | 0,982983 | 1,097327 | 1,08048 |
| ***CCNB1-Hs00259126_m1*** | n.a. | 0,73028 | 0,973601 | 0,99984 |
| ***CCND1-Hs00277039_m1*** | 0,228967 | 0,168937 | 0,870595 | 0,791284 |
| ***CCNE1-Hs00233356_m1*** | 0,330907 | 0,361523 | 0,977716 | 0,922211 |
| ***CD68-Hs00154355_m1*** | 1,157113 | n.a. | 1,008008 | 0,715353 |
| ***CDC25A-Hs00153168_m1*** | 0,181922 | 0,221519 | 1,038806 | 0,850746 |
| ***CDH1-Hs00170423_m1*** | n.a. | 1,051677 | n.a. | n.a. |
| ***CDKN1A-Hs00355782_m1*** | 0,676308 | 0,544368 | 0,993104 | 1,202653 |
| ***CDKN1B-Hs00153277_m1*** | n.a. | 1,321311 | 1,143491 | 1,125299 |
| ***CDKN1C-Hs00175938_m1*** | n.a. | 3,33807 | 0,942056 | 1,287849 |
| ***CDKN2A-Hs00233365_m1*** | 1,086348 | 0,723408 | n.a. | n.a. |
| ***CDKN2C-Hs00176227_m1*** | 1,024564 | 0,761131 | 1,108879 | 1,156401 |
| ***CTSL2-Hs00822401_m1*** | 1,273509 | 0,735404 | 1,098535 | 1,179713 |
| ***CXCR4-Hs00607978_s1*** | 1,293038 | 1,301125 | 0,867247 | 1,119642 |
| ***DAPK1-Hs00234480_m1*** | n.a. | 1,281749 | n.a. | n.a. |
| ***E2F4-Hs00608098_m1*** | 0,657292 | 0,551428 | 1,005242 | 0,914888 |
| ***EGF-Hs00153181_m1*** | 1,938983 | 0,960128 | 1,45266 | 1,667003 |
| ***EGFR-Hs00193306_m1*** | 2,012067 | 1,520151 | 0,953884 | 0,764179 |
| ***EIF4A2-Hs00756996_g1*** | 1,205118 | 1,118178 | 1,210883 | 1,171689 |
| ***EIF4EBP1-Hs00607050_m1*** | 1,057465 | 0,80597 | 0,945586 | 0,994467 |
| ***EIF4E-Hs00854166_g1*** | 0,757494 | 0,576922 | 1,333284 | 0,710903 |
| ***EIF4G1-Hs00191933_m1*** | 0,657823 | 0,444115 | 1,077362 | 0,9656 |
| ***EIF4G2-Hs00154952_m1*** | 0,898961 | 0,564821 | 0,927685 | 1,043377 |
| ***ERBB2-Hs00170433_m1*** | 1,284564 | 1,18043 | 0,886844 | 1,045653 |
| ***FADD-Hs00356603_g1*** | 0,731164 | 0,695613 | 0,962477 | 0,940807 |
| ***FAS-Hs00163653_m1*** | 0,614894 | n.a. | 1,003203 | 1,123376 |
| ***FGF1-Hs00361126_m1*** | 1,117038 | 1,099352 | n.a. | n.a. |
| ***FRAP1-Hs00234522_m1*** | 0,912326 | 0,574262 | 0,831471 | 1,01974 |
| ***GAPDH-Hs99999905_m1*** | 0,807065 | 0,700057 | 1,035464 | 1,061063 |
| ***GATA3-Hs00231122_m1*** | n.a. | 0,895966 | 0,978223 | 1,17192 |
| ***GCN5L2-Hs00221499_m1*** | 0,408177 | 0,393572 | 1,022522 | 0,763595 |
| ***GRB7-Hs00917999_g1*** | 2,648736 | 2,201268 | 0,766473 | 1,096862 |
| ***GUSB-Hs99999908_m1*** | 1,075798 | 1,165129 | 0,979261 | 1,020432 |
| ***HIF1A-Hs00153153_m1*** | 0,663704 | 0,478276 | 0,924153 | 1,041889 |
| ***HOXB13-Hs00197189_m1*** | 0,672798 | 1,242499 | n.a. | 0,909203 |
| ***HSBP1-Hs00833101_s1*** | 0,795126 | 0,581845 | 1,011705 | 1,018924 |
| ***ID1-Hs00357821_g1*** | 0,845788 | 1,132018 | 0,996056 | 0,89303 |
| ***IGF1R-Hs00609566_m1*** | n.a. | 1,816577 | 0,973312 | 0,963926 |
| ***IGF2-Hs00171254_m1*** | 1,777317 | n.a. | 0,7959 | 0,824247 |
| ***IL11-Hs00174148_m1*** | 0,367192 | 0,304443 | 0,866698 | 0,810606 |
| ***IL17RB-Hs00218889_m1*** | 0,397647 | 0,825601 | n.a. | 1,022867 |
| ***IL8-Hs00174103_m1*** | 0,316024 | 1,481572 | 1,085925 | n.a. |
| ***MKI67-Hs00606991_m1*** | n.a. | 0,632095 | 1,195233 | 0,992451 |
| ***MMP11-Hs00171829_m1*** | 0,308881 | n.a. | 0,809558 | 0,946702 |
| ***MMP9-Hs00234579_m1*** | 0,443339 | n.a. | n.a. | 0,7637 |
| ***MTA3-Hs00383033_m1*** | 1,454085 | 0,727502 | 0,995239 | 1,137888 |
| ***MYBL2-Hs00231158_m1*** | 0,357986 | 0,34532 | 0,917154 | 0,89396 |
| ***MYC-Hs00153408_m1*** | 0,96406 | 0,918648 | 0,979989 | 0,923636 |
| ***NCOA3-Hs00180722_m1*** | 0,976746 | 0,934371 | 1,092914 | 1,071106 |
| ***NCOR1-Hs00196920_m1*** | 0,905681 | 0,940617 | 0,865017 | 0,997263 |
| ***NFKB1-Hs00765730_m1*** | 0,879532 | 0,762506 | 0,86344 | 1,011397 |
| ***NFKB2-Hs00174517_m1*** | 1,019594 | 1,722536 | 0,958712 | 0,892518 |
| ***NFKBIA-Hs00153283_m1*** | 0,942518 | 0,819646 | 1,000129 | 1,110556 |
| ***PDGFA-Hs00234994_m1*** | n.a. | 0,711826 | 0,971401 | 1,12861 |
| ***PGR-Hs00172183_m1*** | 1,031379 | 1,006059 | n.a. | n.a. |
| ***PINK1-Hs00260868_m1*** | 1,36 | 1,860072 | 1,153352 | 1,132688 |
| ***PLAU-Hs00170182_m1*** | n.a. | 1,062838 | 1,185319 | 0,933311 |
| ***PSME1-Hs00389209_m1*** | 1,055956 | 0,807009 | 1,049021 | 1,12224 |
| ***PTEN-Hs00829813_s1*** | n.a. | 1,785874 | 1,018979 | 0,707192 |
| ***RAD51C-Hs00427442_m1*** | 0,526807 | 0,626644 | 0,806495 | 0,98 |
| ***RB1-Hs00153108_m1*** | 1,054258 | 0,865124 | 0,924633 | 1,022221 |
| ***RPLP0-Hs99999902_m1*** | 1 | 1 | 1 | 1 |
| ***RPS6KB1-Hs00177357_m1*** | 1,064553 | 0,655639 | 0,965821 | 1,084839 |
| ***SCUBE2-Hs00221277_m1*** | 1,437447 | 1,044023 | 0,673867 | 1,148892 |
| ***SKP2-Hs00180634_m1*** | 0,529384 | 0,89831 | 0,969455 | 0,950118 |
| ***SMAD2-Hs00183425_m1*** | 0,892141 | 0,834718 | 0,965152 | 0,959581 |
| ***STK6-Hs00269212_m1*** | 0,531253 | 0,477648 | 0,916641 | 1,010722 |
| ***TERT-Hs00162669_m1*** | 0,977476 | 0,651477 | 1,078678 | 0,564788 |
| ***TFRC-Hs99999911_m1*** | 0,483168 | 0,275892 | 0,922324 | 0,858931 |
| ***TGFB1-Hs00171257_m1*** | 0,526905 | 0,725677 | 0,938784 | 0,997591 |
| ***TGFBR1-Hs00610319_m1*** | 0,811401 | 0,543034 | 0,935721 | 1,025939 |
| ***TGFBR2-Hs00234253_m1*** | 0,750921 | 1,217553 | 0,911557 | 0,935431 |
| ***VEGF-Hs00173626_m1*** | 1,007255 | 0,553872 | 1,028916 | 0,917575 |

1 x 10^6^ cells were seeded in 10-cm culture dishes, allowed to adhere for 24 h and subsequently treated for 12 h with 300 nM lapatinib or vehicle DMSO. Thereafter, cells were harvested, washed, and used for RNA extraction. Subsequently, LDAs were performed to compare gene expression in lapatinib- vs. vehicle-treated cells. Results were normalized to RPLP0 and expressed as mRNA fold induction in lapatinib-treated versus vehicle-treated cells. Results are means of four separate experiments. *n.a., not available.
