# Supplementary figures and images for: Grb7 Upregulation Is a Molecular Adaptation to HER2 Signaling Inhibition Due to Removal of Akt-Mediated Gene Repression
Source: PLoS One. 2010 Feb 2;5(2):e9024. doi: 10.1371/journal.pone.0009024 (PMC2814867; doi:10.1371/journal.pone.0009024)

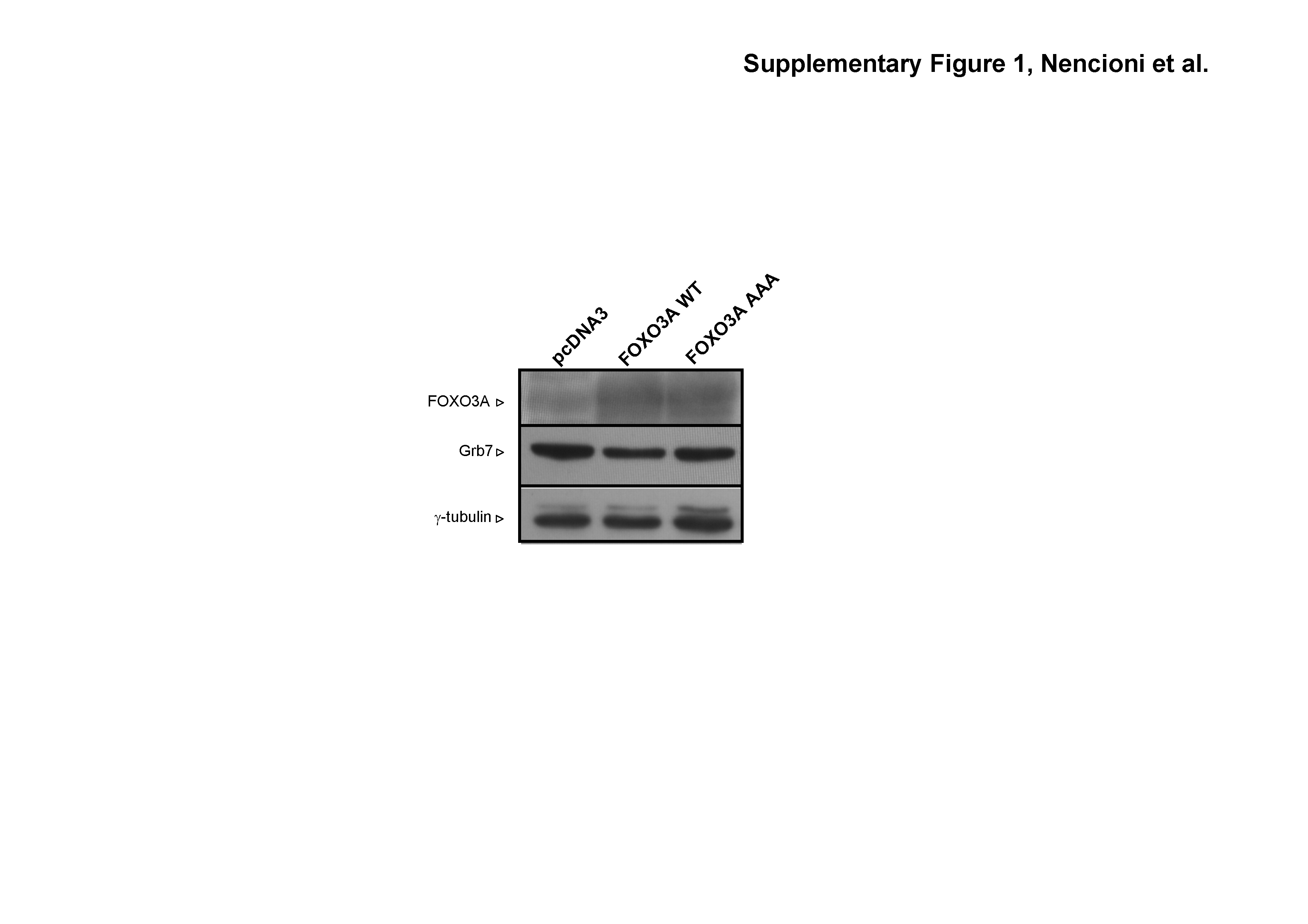

Supplement: Figure S1 — FOXO3A does not affect Grb7 expression in SKBR3 cells. SKBR3 cells were transfected with plasmids encoding WT FOXO3A, FOXO3A AAA or the empty vector (pcDNA3) as a control. Cells were selected for 2 weeks using G418 before being used for cell lysates preparation. FOXO3A, Grb7, and γ-tubulin expression were detected by immunoblotting. (0.70 MB TIF) [file pone.0009024.s002.tif]
